# Systemic amyloidosis with bilateral conjunctival involvement: a case report

**DOI:** 10.1186/s12886-015-0075-2

**Published:** 2015-07-19

**Authors:** Leandro J. Correa, J. Pablo Maccio, Evangelina Esposito, Rodolfo Monti, M. Eugenia Gonzalez-Castellanos, Martin Paradelo, Horacio M. Serra, Julio A. Urrets-Zavalia

**Affiliations:** Department of Ophthalmology, University Clinic Reina Fabiola, Universidad Catolica de Cordoba, Oncativo 1248, Cordoba, 5000 Argentina; Centro Medico de Patologia y Citopatologia, Cordoba, Argentina; CIBICI-CONICET, Faculty of Chemical Sciences, Universidad Nacional de Cordoba, Cordoba, Argentina

**Keywords:** Ocular amyloidosis, Systemic amyloidosis, Red eye, Conjunctival malignancy

## Abstract

**Background:**

Conjunctival amyloidosis is a very rare condition, generally unilateral, and presents mostly as an isolated condition without systemic compromise. Our purpose is to present a new case of systemic amyloidosis with a bilateral conjunctival involvement.

**Case Presentation:**

A 66-years-old caucasian female complaining of conjunctival hemorrhage and chemosis in both eyes for the last five years had been discontinuously treated with topical antibiotics and corticosteroids without any evident improvement. She presented with a pink-yellow infiltration in the inferior conjunctiva of both eyes. Conjunctival biopsy under optical microscopy revealed amyloid deposit, confirmed by Congo red staining. Mucosal biopsy from esophagus and rectus confirmed amyloidosis by Congo red stain. Immunohistochemistry of bone marrow biopsy showed an increased number of plasma cells and an over-expression of light chain kappa subunit. She was treated with corticosteroids and lubrication with an improvement of symptoms. Ocular lesions remained stable after a follow-up of 3 years.

**Conclusions:**

Conjunctival amyloidosis is a rare entity that may be overlooked, and should be differentiated from chronic conjunctivitis and conjunctival malignancies. Although it presents most frequently as a local process, a systemic involvement should always be ruled out.

## Background

Amyloidosis is a rare group of disorders characterized by the deposition of insoluble fibrillar proteins in a β-pleated sheet configuration, known as amyloid, within the extracellular and perivascular space, as the consequence of a wrong folding process of normally soluble proteins, and may affect virtually any organ or tissue of the body [1–5].

Amyloidosis disorders are classified into three major forms: local, systemic, and hereditary systemic amyloidosis [2]. The acquired forms may be primary (immunologic) and secondary (reactive) [2]. In primary amyloidosis (AL) deposits contain immunoglobulin light chains, usually lambda and kappa monoclonal types, resulting from the abnormal production by plasmocytes in the bone marrow [2, 6]. Secondary amyloidosis (AA) is formed from serum protein A, an acute phase reactant protein that is synthesized in response to longstanding inflammation [7]. In hereditary amyloidosis protein deposits are constituted by a mutant form of the transport protein transthyretin [8].

Ocular amyloidosis occurs most frequently as the local deposition of amyloid in the same place where it originates or, rarely, may be part of a systemic disorder [3, 9]. Amyloid may accumulate in the eyelid, conjunctiva, cornea, vitreous, or anterior orbit [3, 10].

Conjunctival amyloidosis is one of the most common forms of ocular involvement, mostly occurring as a local deposition of amyloid and rarely in association with systemic involvement [3, 10–15]. Due to the rarity of conjunctival amyloidosis, its diagnosis can often be overlooked or confused with other conditions that may affect the conjunctiva. It is more commonly observed in middle-aged adult patients, presenting as an inflammatory or malignant conjunctival entity [3, 11]. Besides, conjunctival malignant lesions can lead to amyloidosis and they should be ruled out [3, 12].

The purpose of our study is to report a new case of systemic amyloidosis with bilateral conjunctival involvement.

## Case presentation

A 66-year-old Caucasian woman complaining of conjunctival hemorrhage and chemosis in both eyes for the last five years had been discontinuously treated with topical antibiotics and corticosteroids without any evident improvement.

**Fig. 1:**
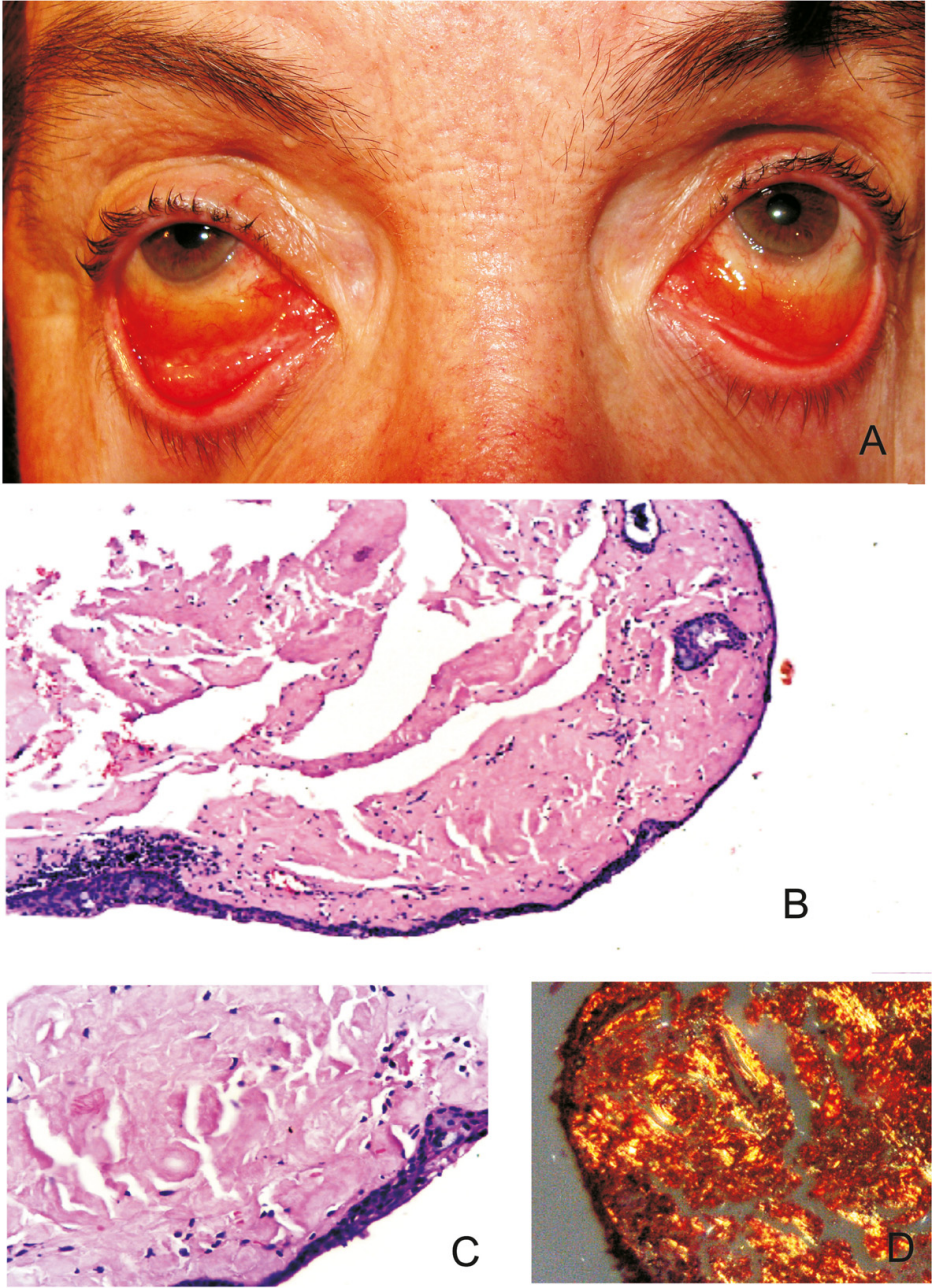
(**a**)Photograph of the patient showing in both eyes diffuse, yellow-pink diffuse mass extending from the inferior bulbar conjunctiva to the lower fornix with areas of waxy-yellow condensations, prominent intrinsic vessels and subconjunctival hemorrhage. **b** Optical microscopy of conjunctival biopsy shows an amorphous eosinophilic material with mild mono and polymorphonuclear inflammatory infiltrate and hemorrhagic suffusion; (H&E; original magnification x100). **c** A higher magnification shows the amorphous material replacing the normal stroma of the conjunctiva; (H&E; original magnification x400). **d** Polarization microscopy of conjunctival specimen stained with Congo red shows a characteristic red-green birefringence and dichroism of amyloid

At the time of her referral, best-corrected visual acuity was 20/25 in both eyes. Biomicroscopy showed significant bilateral and rather symmetric edema of the inferior bulbar and lower fornix conjunctiva with areas of pink-yellow condensation and hemorrhages associated with follicular reaction (Fig. 1a). A conjunctival swap was obtained to rule out infection by common antibiotic resistant bacteria or Chlamydia spp. As direct fluorescent antibodies for Chlamydia spp were positive, she was put under treatment with oral doxycycline 200 mg/day and topical azithromycin 3 times/day for 20 days, without any improvement. Conjunctival biopsy was undertaken and optical microscopy revealed amorphous eosinophilic material positive for hematoxyline-eosin stain, with mild mono and polymorphonuclear inflammatory infiltrate and hemorrhagic suffusion (Fig. 1b and 1c). After Congo red staining, the samples showed a red-green birefringence and dichroism under polarized light microscopy consistent with amyloidosis (Fig. 1d). No evidence of malignancy was observed.

**Fig. 2:**
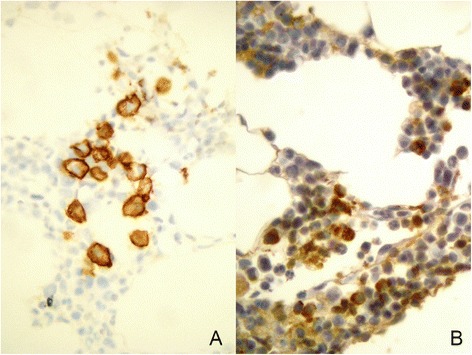
Immunohistochemistry of bone marrow biopsy. **a** Staining with specific antibodies for CD 138 show increased number of plasma cells (in brown; x400). **b** Over-expression of immunoglobulin light chain kappa subunit (in orange-brown; x400)

A complete systemic evaluation was carried out. Liver and heart function evaluation was normal. Immunofixation of blood samples revealed polyclonal hypergammaglobulinemy, and no monoclonal bands were observed by immunofixation of urine sample. Mucosal biopsy from esophagus, and rectus confirmed amyloidosis by the Congo red staining (not shown). In order to rule out multiple myeloma or plasma cells dyscrasia, a bone marrow biopsy was obtained showing a positive expression of CD 138+ (Fig. 2a), a well known marker for identification and quantification of plasma cells in bone marrow and other tissues, as well as an increased population of plasma cells with over-expression of the light chain kappa subunit (Fig. 2b).

The patient was treated with topical lubrication and the ocular lesions of both eyes remained stable after a follow-up of 3 years. She continues with periodical clinical controls to assess the eventual manifestation and progression of the systemic disease.

## Discussion

Conjunctival amyloidosis, predominantly affecting middle-aged adults, usually begins at the fornices, spreading to the bulbar and palpebral conjunctiva [3]. This progression appears to affect more deeply the palpebral than the bulbar conjunctiva [3, 16]. In our patient, a bilateral diffuse pink-yellow infiltration with hemorrhagic areas was found throughout the lower bulbar conjunctiva without affecting the palpebral function and position, in contrast with the findings of Demirci et al [4] that observed a unilateral involvement in all of their six cases.

Associated spontaneous conjunctival hemorrhages, as observed in our patient, are a common finding that may be explained by the fact that amyloid infiltration of vessel walls induces rigidity and disruption of conjunctival vessels [3]. A complete systemic evaluation to discard a primary systemic form of the disease is recommended [10] as conjunctival amyloidosis might be an early manifestation of systemic amyloidosis [3]. In our case, the initial presentation of conjuctival amyloidosis led to the systemic findings that were asymptomatic. Only five cases have been reported as systemic amyloidosis with conjunctival involvement [3, 12–15].

Even though the condition may be clinically suspected, a tissue biopsy of the involved organ should be taken to confirm the diagnosis. In systemic disease the sampling can be performed directly in the organ or tissue affected, or in a clinically uninvolved site such as subcutaneous fat, minor salivary glands or rectal mucosa [1, 3]. Furthermore, testing for monoclonal protein population should be determined by serum and urine protein electrophoresis and immunofixation [1]. In our patient, immunofixation of blood samples only revealed polyclonal hypergammaglobulinemy, and rectal and esophageal mucosal biopsies confirmed the diagnosis of systemic amyloidosis. In addition, immunohistochemical studies of bone marrow biopsies showed an increased population of plasma cells with the amyloidogenic overexpression of the subunit kappa.

There is still no consensus on the management of conjunctival amyloidosis and none of the available treatments are radical. Some authors recommend conservative treatment using lubricants and topical anti-inflammatories [3]. Others believe that a more aggressive treatment should be done in order to prevent progression or recurrence [17]. Cryotherapy, [18] radiotherapy, [19] surgical debulking, [20] and mucous membrane graft implantation, [21] are among the techniques that could be used. A conservative topical treatment was offered to our patient, as lesions remained stable over time and the patient referred no major ocular complaints.

## Conclusions

Conjunctival amyloidosis is a rare entity that may be overlooked and should be differentiated from chronic conjunctivitis and conjunctival malignancies. Although commonly a local process, a systemic involvement should always be ruled out.

## Consent

Written informed consent was obtained from the patient for publication of this Case report and any of the accompanying images. A copy of the written consent is available for review by the Editors of BMC Ophthalmology.
